# Induced HBs antigenemia in healthy adults after immunization with two different hepatitis B recombinant vaccines

**Published:** 2010-12-01

**Authors:** Masoud Ziaee, Alireza Saádatjoo, Malihe Mohamadpour, Mohammad Hasan Namaei

**Affiliations:** 1 Infectious Disease Ward, Birjand University of Medical Sciences, Birjand, IR Iran; 2 Birjand University of Medical Sciences, Birjand, IR Iran; 3 Bahar Rehabilitation Institute, Birjand, IR Iran; 4 Birjand University of Medical Sciences, Birjand, IR Iran

**Keywords:** Hepatitis B vaccine, Hepatitis B Surface Antigens

## Abstract

**Background and Aims:**

Currently, vaccination is the most effective protective tool against hepatitis B virus infection. Some studies have shown that positive results for a hepatitis B virus surface antigen (HBsAg) test may be seen after vaccination.

**Materials and Methods:**

In this clinical trial study, 62 healthy adult volunteers were randomly assigned to receive either the Engerix-B or the Hepavax-Gene hepatitis B recombinant vaccine. Blood samples were drawn 1, 3, and 5 days after vaccination and were tested for HBsAg using two different ELISA kits (Behring and Mega).

**Results:**

HBsAg was positive in 5, 3, and 2 participants of the Engerix-B group in the 1st, 3rd, and 5th days after vaccination, respectively, using the Behring ELISA kit the test was positive in only one subject in the Hepavax-Gene group, on the 5th day after vaccination. No positive result was seen in any groups when the Mega ELISA kit was used to test the specimens.

**Conclusions:**

Our results showed transient HBsAg antigenemia after vaccination against hepatitis B. This condition depends on the type of vaccine and the HBsAg diagnostic test.

## Introduction

Hepatitis B, the most serious type of viral hepatitis, is a major health problem throughout the world. According to a WHO report, around 2 billion people are infected with the hepatitis B virus (HBV) in the world, and about 600,000 people die annually due to its acute or chronic consequences. There are more than 350 million HBV carriers in the world [1]. People who are in continuous contact with blood and bodily fluids are the most likely to contract hepatitis B. High-risk populations include dialysis patients, health-care workers, infants born to mothers infected with HBV, and intravenous drug abusers [2].Vaccination against hepatitis B plays an important role in the prevention of HBV infection in high-risk population. An effective vaccination can prevent HBV infection in 95% of immunized individuals as well as the hepatocellular carcinoma in the children of chronic HBV infection [3].A predictable group for hepatitis B contamination as high-risk population was the first target for vaccination in the United States after the first development of hepatitis vaccine in 1981. In contrast, the vaccine was not very successful and the rate of hepatitis B continued to grow. Therefore, in 1991 the Centers for Disease Control suggested vaccination for both high-risk adolescents and all newborns, and subsequently the international recommendation (by WHO) was to vaccinate all infants against hepatitis B by 1997 [4]. This strategy led to a decrease in the incidence of hepatitis B worldwide [3][5][6].Some studies have reported positive results of HBsAg tests after hepatitis B immunization in infants [7][8][9] as well as adolescents [5][10][11]. False positive HBsAg results after vaccination can lead to the misdiagnosis of being infected with hepatitis B for healthy individuals, causing unnecessary concern and treatments. A careful diagnosis of HBV is even more important in blood donors. Dow et al. [12] showed that hepatitis B vaccination can lead to a misdiagnosis in blood donors in the week after vaccination. Otag [11] Emphasizes that the period of antigenemia could extend for up to one month.The aim of this study was to compare the incidence of positive results of HBsAg tests after vaccination with two different hepatitis B recombinant vaccines.

## Materials and Methods

This clinical trial study was conducted on 62 healthy medical students who were at the beginning of their clinical courses and needed to be vaccinated against HBV. They were between 20 and 22 years old and tested negative for HBc antibody and had no history of HBV vaccination, jaundice, or blood or blood-derived-products transfusion during the past 3 months. The study protocol was approved by the research ethics committee of the Birjand University of Medical Sciences, and all participants signed an informed-consent form for participation. Subjects were assigned into two vaccination groups by simple randomization. The two types of vaccines were Hepavax-Gene (manufactured by Green Cross Vaccine Corp., Seoul, Korea) and Engerix-B (manufactured by GlaxoSmithKline, Rixensart, Belgium). There were 32 and 30 subjects in the Hepavax-Gene and Engerix-B groups, respectively. A full dose of vaccine was administrated intramuscularly for all subjects as the first dose of vaccination against HBV. All subjects were vaccinated 1 and 6 months after the first vaccination. Blood samples were drawn 1, 3, and 5 days after the first dose of vaccination and tested for HBsAg by two different ELISA kits (Behring, Marburg, Germany; and Mega, Los Angeles, California, USA). All tests were performed by one person in the same setting. The immunization status of all participants was evaluated 3 months after vaccination by testing for HBsAb using Behring (Marburg, Germany) anti-HBs ELISA kits. The results are expressed as mean ± standard deviation (SD) for quantitative variables and percentages for categorical variables. Categorical variables were compared with a Fisher exact test. P values of 0.05 or less were considered statistically significant. All statistical analyses were performed using SPSS version 15.

## Results

Of the 62 participants, 36 (58.1%) were female. Half of the women were in the Engerix-B group and half were in the Hepavax-Gene group. Positive results for the HBsAg test in the 1st, 3rd, and 5th day after vaccination with the two types of vaccines were different when the Behring ELISA kit was used. In the Engerix-B group, 5 (16.7%), 3 (10%), and 2 (6.7%) people had positive results 1, 3, and 5 days after vaccination, respectively (Table 1). In the Hepavax-Gene group, no one had positive results in Days 1 and 3, and only 1 (3.1%) person tested positive 5 days after vaccination. There was a statistically significant differences between the two vaccination groups in the prevalence of false positive results of HBsAg on day 1 (Table 2).

No positive results were observed in any person in either vaccination group when the Mega ELISA kit was used to test for HBsAg in the blood specimens.All subjects were tested for HBsAb 3 months after completing their vaccination. Additionally, all individuals in both groups had a protective antibody level of more than 10 IU/ml.

**Table 1:** Result summary of subjects with false positive results of hepatitis B surface antigen test

**Vaccine type**	**Patient No.**	**Age**	**Sex**	**Days after Vaccination**	**HBsAg Test**
Engerix-B	1	21	female	1	Positive
				3	Negative
				5	Negative
	2	20	female	1	Positive
				3	Negative
				5	Negative
	3	22	female	1	Positive
				3	Positive
				5	Negative
	4	21	male	1	Positive
				3	Positive
				5	Negative
	5	22	male	1	Negative
				3	Positive
				5	Negative
	6	20	male	1	Negative
				3	Negative
				5	Positive
	7	21	male	1	Negative
				3	Negative
				5	Positive
	8	22	female	1	Positive
				3	Negative
				5	Negative
Hepavax-Gene	1	21	male	1	Negative
				3	Negative
				5	Positive

**Table 2:** Comparison of the incidence of HBsAg false positive results between the two vaccine groups during the first 5 days after vaccination

		**Vaccine group a**	**p-Value**
**Hapavax-Gene(No.=32)**	**Engerix-B (No.=30)**
**After 1 day**	0	5 (16.7%)	0.02 b
**After3 day**	0	3 (10%)	0.07
**After5 day**	1 (3.1%)	2 (6.7%)	0.52

^a^ Number of individuals with a positive result on the HBsAg test (percentage of N)

^b^ Differences between the two groups were statistically significant.

## Discussion

HBsAg is the most common serological marker for the diagnosis of hepatitis B. Contrary to recent studies [11][12], primary research after the development of plasma-derived HBV vaccine did not reveal postvaccination HBs antigenemia [13]. Our study showed transient HBs antigenemia after administration of vaccinating against hepatitis B. The results of this study showed that different types of hepatitis B vaccines can result in different rates of false positive results for an HBsAg test. As shown in table 1, in the Engerix-B group, 8 (26%) people tested positive for HBsAg during 5 days after vaccination, but Hepavax-Gene caused antigenemia in only 1 subject (3.1%). In a similar study, Otag [11] found different rates of HBs antigenemia caused by different hepatitis B vaccines as well, but because the sample size of her study was low, she was not able to perform any statistical analysis.Our results indicate that different HBsAg ELISA kits detect antigenemia differently. In this study, the Behring ELISA kit found 8 (26.7%) false positive HBsAg cases in the Engerix-B group, whereas the Mega ELISA kit did not detect any antigenemia. Dow et al. [12] used 3 different HBsAg assays with 8 volunteers to test antigenemia in the days after immunization. Similar to our study, there were differences between the results of the HBsAg assays. Of the 8 HBsAg-positive subjects in the Engerix-B group, the development of antigenemia occurred at different periods of time. Specifically, 5, 1, and 2 participants developed antigenemia at 1, 3, and 5 days after vaccination, respectively. There was one positive antigenemia in the Hepavax-Gene group, which was found on Day 5. Some studies have reported different times for the onset of antigenemia [14]. Based on the results, all subjects with an onset of antigenemia in the first 3 days after vaccination had a negative HBsAg result on Day 5. The clearance period (i.e., the time between the appearance of HBsAg and when it is undetectable) in our study ranged between 2 and 4 days. In Otag's study, antigenemia disappeared within 3 days [11]. Some studies have reported prolonged antigenemia in neonates or hemodialysis patients, even after several weeks [9][15]. In our study, there was no difference in the acquisition of immunity between the Engerix-B and Hepavax-Gene groups after 3 months of follow-up.

## Conclusion

Our results indicate that HBs antigenemia may occur after vaccination against hepatitis B. This positive HBsAg result can vary by the type of vaccine or across different HBsAg ELISA kits. Therefore, health-care workers should be informed about possible HBs antigenemia after vaccination against hepatitis B. Still, there is no difference in immunization between subjects with and without false positive HBsAg results.
